# Extracorporeal mechanical support and aspiration thrombectomy in treatment of massive pulmonary embolism: a case report

**DOI:** 10.5935/0103-507X.20220342-en

**Published:** 2022

**Authors:** João Valente Jorge, Catarina A Barreiros, Doroteia Silva, Rita Calé, João Miguel Ribeiro

**Affiliations:** 1 Anesthesiology Department, Hospital Universitário Santa Maria, Centro Hospitalar Universitário Lisboa Norte - Lisboa, Portugal.; 2 Intensive Care Department, Hospital Universitário Santa Maria, Centro Hospitalar Universitário Lisboa Norte - Lisboa, Portugal.; 3 Cardiology Department, Hospital Garcia de Orta - Almada, Portugal.

**Keywords:** Pulmonary embolism, Cardiac arrest, Extracorporeal membrane oxygenation, Thrombectomy

## Abstract

Acute massive pulmonary embolism is the most serious presentation of venous thromboembolism that can ultimately cause obstructive shock, leading to cardiac arrest and death. In this case report, the authors present a case of a 49-year-old female who successfully recovered from a massive pulmonary embolism with the combined use of venoarterial extracorporeal membrane oxygenation and pulmonary aspiration thrombectomy, with no complications from these procedures. Although evidence of benefit from mechanical support has not been established for patients with massive pulmonary embolism, the implementation of extracorporeal cardiocirculatory support during resuscitation may allow improvement of systemic organ perfusion and better chance of survival. Recent guidelines from the European Society of Cardiology state that venoarterial extracorporeal membrane oxygenation in combination with catheter-directed treatment may be considered for patients presenting with massive pulmonary embolism and refractory cardiac arrest. The use of extracorporeal membrane oxygenation as a stand-alone technique with anticoagulation remains controversial, and additional therapies, such as surgical or percutaneous embolectomy, must be considered. Since this intervention is not supported by high-quality studies, we believe it is important to report real-world successful cases. With this case report, we illustrate the benefit derived from resuscitation assisted by extracorporeal mechanical support and early aspiration thrombectomy in patients with massive pulmonary embolism. Additionally, it emphasizes the synergy that derives from integrated multidisciplinary systems for providing complex interventions, of which extracorporeal membrane oxygenation and Interventional Cardiology are clear examples.

## INTRODUCTION

Acute massive pulmonary embolism (PE) is the most serious presentation of venous thromboembolism.^(1-3)^ In its most severe form, it causes obstructive shock that can culminate in cardiac arrest and sudden death. Early administration of thrombolysis might reverse some cases of severe obstructive shock and even cardiac arrest, but significant mortality still persists.^(3)^ Although evidence of benefit from mechanical support has not been established for patients with massive PE and cardiac arrest, the implementation of extracorporeal cardiocirculatory support during resuscitation may allow improvement of systemic organ perfusion and better chance of survival. Recent guidelines from the European Society of Cardiology (ESC) state that venoarterial extracorporeal membrane oxygenation (VA-ECMO) in combination with catheter-directed treatment may be considered for patients presenting with massive PE and refractory cardiac arrest.^(4)^ In this report, the authors present a case of cardiac arrest secondary to massive PE that was successfully treated with extracorporeal cardiopulmonary resuscitation and direct aspiration thrombectomy by a multidisciplinary team from two tertiary referral hospitals.

## CASE REPORT

**Figure 1:**
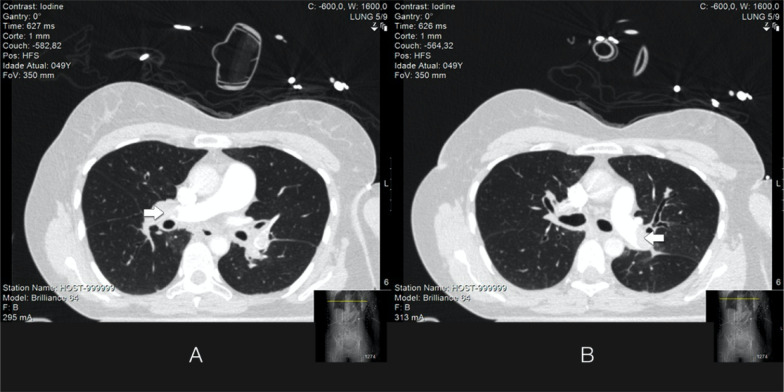
Computed tomography pulmonary angiogram showing major central pulmonary thrombus (arrows) in the right (A) and left (B) pulmonary arteries.

**Figure 2:**
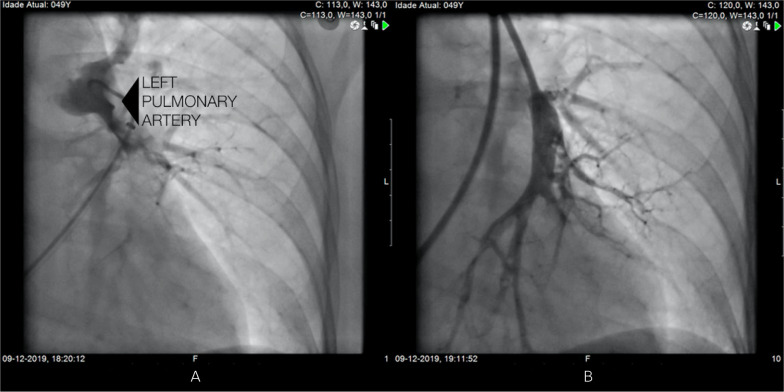
Angiography of the left pulmonary artery (arrow) before (A) and after (B) pulmonary thromboembolectomy.

**Figure 3:**
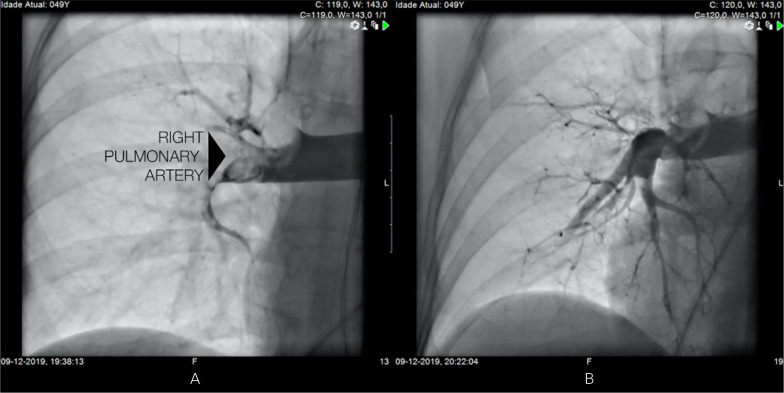
Angiography of the right pulmonary artery (arrow) before (A) and after (B) pulmonary thromboembolectomy.

A 49-year-old female was admitted to our intensive care unit (ICU) during advanced resuscitation efforts following sudden cardiac arrest. The patient had a fall while walking, with no apparent prodrome symptoms, at 8:10 AM. Minor head trauma and loss of consciousness occurred. The prehospital emergency team arrived at the scene three minutes later. Spontaneous recovery of consciousness was soon followed by syncope, and tracheal intubation and assisted ventilation were performed. Pulseless electrical activity evolved, and immediate Advanced Cardiac Life Support began, with return of spontaneous circulation (ROSC) after four minutes. At 8:54 AM, a new episode of cardiac arrest with pulseless electrical activity occurred, immediately followed by resuscitation maneuvers with mechanical chest compression with the LUCAS3™ device; ROSC was achieved 28 minutes later. During this period, she was transported to the hospital and directly admitted to our ICU. Transthoracic echocardiogram immediately showed severe dilation of the right ventricle and signs of pressure overload. A weight-adjusted bolus injection of nonfractional heparin was administered. Eventually, she had ROSC, but a third episode of cardiac arrest occurred at 9:22 AM. This time, it was decided to proceed with extracorporeal cardiopulmonary resuscitation, assuming a major contraindication for thrombolysis due to recent head trauma. Extracorporeal flow support, with a femoro-femoral VA-ECMO configuration, was effectively implemented at 10:16 AM (Getinge CardioHelp device). In the next few minutes, a computed tomography pulmonary angiogram was performed that established a definitive diagnosis of PE involving both pulmonary arteries, with further extension to the lobar segments of both sides (Figure 1). Brain tomography revealed no traumatic injury. Direct thrombectomy was immediately considered, and a specialized Interventional Cardiology team from another urban hospital was activated. This multidisciplinary team performed a percutaneous aspiration thrombectomy. A 10-Fr Flexor® Check-Flo long sheath (Cook Inc, Bloomington, Indiana) was placed with the tip in the main right or left pulmonary artery. Through the sheath, a Penumbra Indigo aspiration system CAT8 XTORQ (Penumbra Inc, Alameda, California) was advanced into segmental, lobar, or main pulmonary arterial thrombus, and continuous aspiration was performed (Figures 2 and 3). A Penumbra Indigo System Separator SEP8 device (Penumbra Inc) was used through the aspiration catheter to facilitate clot aspiration by preventing the catheter from clogging the extensive thrombus. Postprocedure angiography showed a marked reduction in proximal thrombi and improvement in bilateral perfusion of the pulmonary arterial system. After this thrombectomy procedure, the mean pulmonary artery pressure dropped from 41mmHg to 13mmHg, reversing pulmonary hypertension. For the next few hours after thrombectomy, the patient remained hemodynamically stabilized under VA-ECMO support and norepinephrine. Transthoracic echocardiogram revealed resolution of right ventricle dilatation, with normalization of contractility indices. Venoarterial extracorporeal membrane oxygenation support was maintained for 37 hours, with no major adverse events detected; an anticoagulation strategy with nonfractional heparin titrated to an activated partial thromboplastin time (aPTT) ratio of 1.8 was maintained, and ECMO was explanted under our protocol. Following stabilization, warfarin was prescribed. Prothrombotic investigations established a history of recent minor gynecological procedures and regular consumption of oral contraceptives. Additionally, heterozygosity for the prothrombin gene mutation (PT20210A mutation) was documented by DNA analysis, along with functional deficiency of C protein and antithrombin III (41% and 54%, respectively). Furthermore, her father had sudden death of unknown cause, and her daughter had a diagnosis of PE at age 16, with primary thrombophilia associated with double heterozygosity mutation for the methylenetetrahydrofolate reductase gene. Genetic consultation was prescribed for both members of the family. Immediately after admission, a Glasgow Coma Scale score of 8 was diagnosed, and targeted temperature management was implemented. Repeated imaging evaluation revealed hypoxic-ischemic sequelae involving relative undifferentiation of the basal ganglia, confirmed by magnetic resonance. Seizure activity was repeatedly excluded. Eventually, the patient had partial recovery of mental status, with signs suggestive of man-in-the-barrel syndrome. An intensive rehabilitation program was associated with improvements in physical and mental performance, but the patient remained dependent on personal care. At discharge, on Day 51 after admission, the patient had a Cerebral Performance Category score of 3. She was able to follow commands, interact with the observer, use simple words and cooperate with simple tasks, such as physical rehabilitation and eating. At present, she is still recovering in an integrated continuous care unit.

## DISCUSSION

Acute PE is a major cause of cardiac arrest and mortality. Patients with PE have an increased chance of survival if right ventricle unloading and end-organ perfusion can be restored in a short time frame. Thrombolysis during resuscitation is one of the strategies recommended for patients who present with cardiac arrest and a high suspicion of massive PE. Catheter-directed therapy is another approach that is recommended to rapidly restore pulmonary artery patency.^(5)^ On the other hand, extracorporeal circulatory support (or ECMO) can ameliorate organ perfusion during cardiac arrest. In fact, recent guidelines from the ESC established a level IIb recommendation for the use of ECMO in combination with surgical embolectomy or catheter-directed therapy (rather than thrombolysis) for the treatment of patients presenting with refractory circulatory collapse or cardiac arrest.^(4)^ Our therapeutic choice was consistent with these recently published European guidelines. The use of ECMO as a stand-alone technique with anticoagulation remains controversial, and additional therapies, such as surgical or percutaneous embolectomy, must be considered.^(6)^ Since this intervention is not supported by high-quality studies, we believe it is important to report real-world successful cases. We further believe that this report describes the first patient treated with this strategy in our country. Questions about aspiration thrombectomy are also relevant. Several techniques have been described for mechanical disruption of the pulmonary thrombus, all of which aim to relieve obstruction quickly and restore pulmonary blood flow, reduce pulmonary vascular resistance and right ventricle overload and increase cardiac output.^(7,8)^ Catheter-directed therapies for acute PE proved to be safe and effective in small registries and noncontrolled cohort studies.^(9)^ The choice for a percutaneous approach with aspiration thrombectomy with the Penumbra Indigo CAT8 device, in detriment of a surgical thrombectomy, was made in our patient because we assumed it to be the best approach to restore artery patency. Recently, data from the EXTRACT-PE trial have been revealed to the scientific community.^(10)^ This trial, a multicenter investigation conducted under an investigational device exemption from the US Food and Drug Administration, demonstrated that aspiration thrombectomy for acute PE with the Indigo Aspiration System met primary efficacy and safety endpoints. It was shown to be less invasive and to reduce the right-to-left ventricle diameter ratio in less than 48 hours. Furthermore, it provides flexibility for placement in segmental branches of the pulmonary artery; although luminal diameter limits the volume of clot aspirated, it is large enough to minimize clot burden. This leads to hemodynamic and clinical improvement, alleviating the right ventricle pressure overload and restoring contractility. This chain of events was easily demonstrated in our patient. Rapid hemodynamic improvement allowed the explantation of ECMO support on day two after admission, with resolution of all organ perfusion indices. We are aware that extracorporeal mechanical support is not widely available for implementation during resuscitation (ECPR - extracorporeal cardiopulmonary resuscitation). Therefore, it is important to define a referral network that allows the prehospital emergency team to rapidly access highly equipped hospitals that can easily implement ECPR for patients with refractory out-of-hospital cardiac arrest. Interventional cardiology is also essential and a key player in this pathway for survival. In our country, we are developing a strong referral network, and we hope that this kind of organization model will lead to improvement in outcomes of more patients. However, we must recognize that ECPR programs and direct access to interventional cardiology are not easy to implement. Our case report also demonstrates the potential for multidisciplinary collaboration, including the recruitment of medical teams from more than one hospital. This paradigm should allow for better equity when dealing with patient access to highly differentiated medical interventions.

## CONCLUSION

With this case report, we illustrate the benefit derived from resuscitation assisted by extracorporeal mechanical support and early aspiration thrombectomy in patients with massive pulmonary embolism. Additionally, it emphasizes the synergy that derives from integrated multidisciplinary systems for providing complex interventions, of which extracorporeal membrane oxygenation and interventional cardiology are clear examples.
